# VURD Syndrome Managed by Pyelostomy

**DOI:** 10.1100/tsw.2004.80

**Published:** 2004-06-28

**Authors:** Charles J. Rosser, Sam Auringer, R. L. Kroovand

**Affiliations:** Department of Urology, Bowman Gray School of Medicine Wake Forest University, Winston-Salem, North Carolina, USA

## Abstract

We report a case of VURD syndrome in a three day old neonate who was diagnosed with hydronephrosis on a prenatal ultrasound. Severe tortuosity and dilation of the upper urinary tracts in the presence of progression of hydronephrosis or a persistently elevated creatinine may favor a proximal urinary diversion rather than primary valve ablation or cutaneous vesicostomy. Because of a persistently elevated serum creatinine, a nonfunctioning kidney with grade 4/5 vesicoureteral reflux and worsening contralateral hydronephrosis despite lower tract drainage, a left cutaneous pyelostomy was performed, contralateral to the kidney involved with VURD. Postoperatively the serum creatinine stabilized at 1.0 mg/dl and decreased to 0.3 mg/dl at one month of age.

## INTRODUCTION

Rittenberg et al. described in 119 patients the genitourinary abnormalities of the posterior urethral valve, unilateral vesicoureteral reflux, and renal dysplasia as the VURD syndrome.[1] We report a case of VURD syndrome in a three day old neonate.

## CASE REPORT

A pregnant twenty-four year old white female with no known medical disease was found to have a fetus who had oligohydramnios and mild bilateral hydronephrosis at 30 weeks gestation. After subsequent pre-term labor, the patient was born at 32 weeks by vaginal delivery and weighed 2.2 kg with no abdominal masses appreciated on physical exam. A postnatal ultrasound demonstrated severe bilateral hydronephrosis. Laboratory evaluation included a urinalysis which was normal and serum electrolytes which were normal except for a creatinine of 1.8 mg/dl. A voiding cystourethrogram demonstrated grade 4 vesicoureteral reflux on the right, a thickened bladder wall, and posterior urethral valves (Fig.1) and (Fig. 2). A 5 French feeding tube was inserted to drain the bladder.

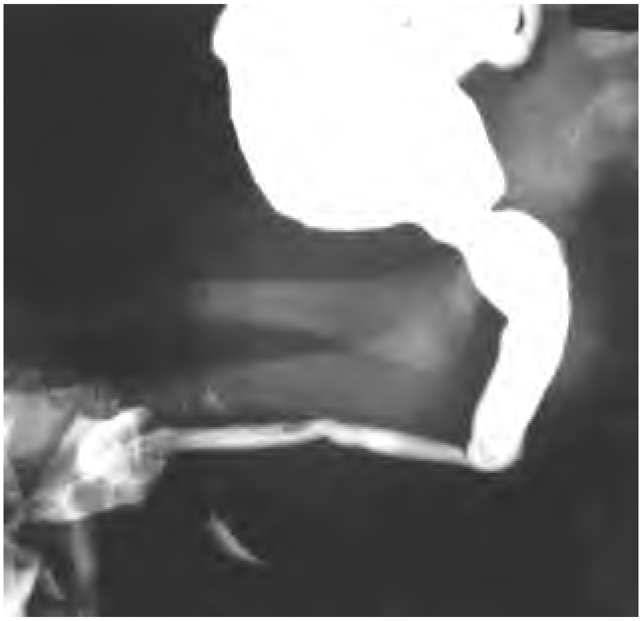

**FIGURE 1.** Voiding cystourethrogram demonstrating posterior urethral valves and dilation of proximal urethra.

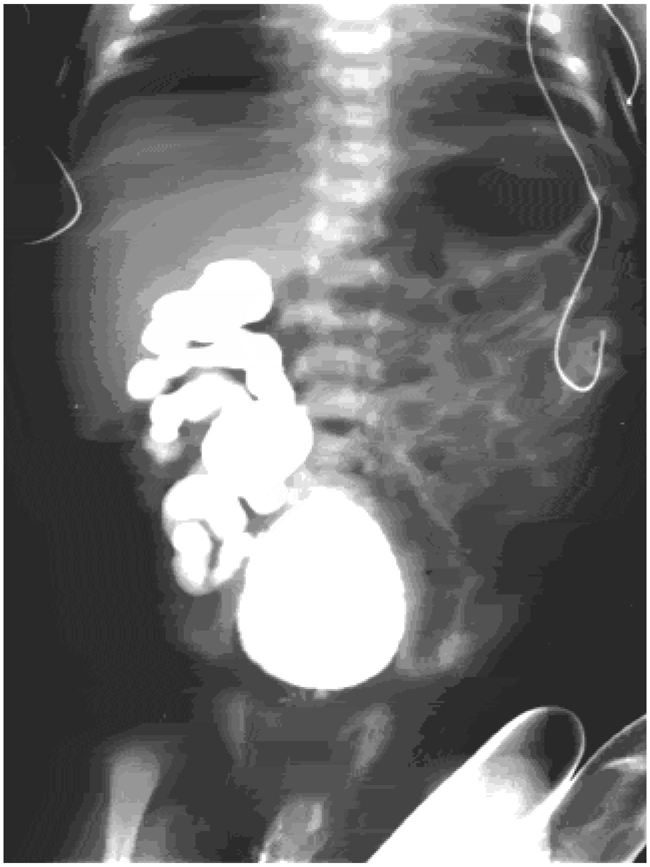

**FIGURE 2.**

Twenty four hours after the insertion of the urinary catheter, the patient's repeat creatinine was 1.7 mg/dl. A repeat ultrasound showed stable hydronephrosis on the right, increase hydronephrosis on the left, and a decompressed bladder. A renogram demonstrated no function on the right and good uptake of the radioactive isotope on the left with delayed excretion even after the administration of Lasix (Fig.3).

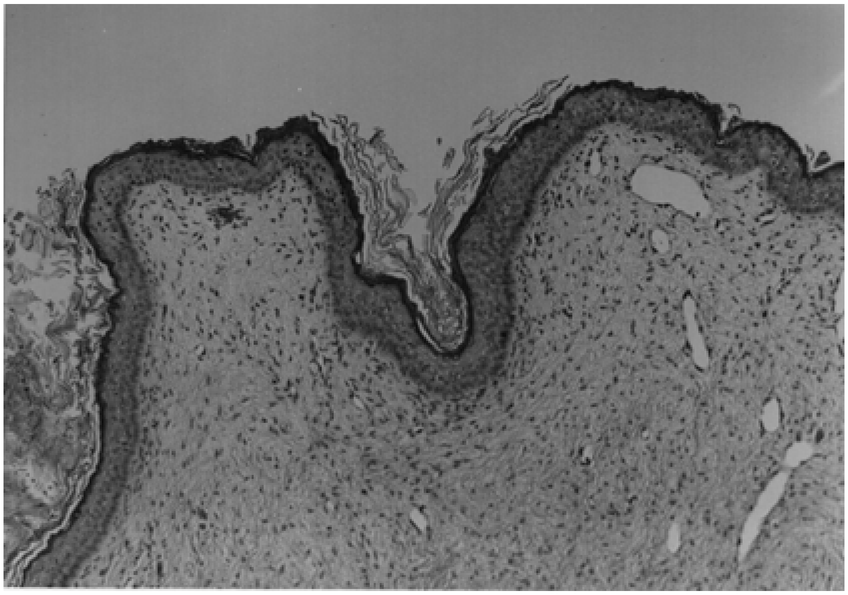

**FIGURE 3.** Microscopic examination of the bladder illustrating hypertrophy of the muscularis propia from chronic obstruction.

Following a left percutaneous nephrostomy for suspected supravesical obstruction, the patient's creatinine decreased to 1.4 mg/dl. An antegrade nephrostogram with an open urinary catheter drainage demonstrated non-obstructed flow into the bladder (Fig.4). Due to the persistently elevated creatinine and the increased risk of infections with prolonged intubated urinary drainage, the patient was taken to the operating suite for a left cutaneous pyelostomy and left renal biopsy. The biopsy showed immature kidney with no specific diagnostic histopathologic changes. Postoperatively, the patient's creatinine stabilized at 1.0 mg/dl. At the patient's one month follow-up, serum creatinine was noted to be0.3 mg/dl.

## DISCUSSION

Prenatal ultrasonography permits in utero diagnosis of hydronephrosis prior to clinical evidence of an obstructive uropathy during the postnatal period.[2,3] Sonography is repeated postnatally to confirm the presence of hydronephrosis. A voiding cystourethrogram (VCUG) is required to complete the evaluation of hydronephrosis.[2] Vesicoureteral reflux as demonstrated by VCUG is present in approximately 50% of the patients with posterior urethral valves.[2] Specifically, unilateral reflux was present in 23.9% with the left ureter affected twice as often as the right.[4] Reflux in association with posterior urethral valves implies a worse prognosis for the involved kidney.[5] However, the nonrefluxing renal unit is protected from the increase hydrostatic pressure in the collecting system by the "pop-off" effect of the reflux ingrenal unit.[3-6] Twenty five to thirty percent of VURD patients have low grade vesicoureteral reflux (grade 1-3) the consequences of high intravesical pressures. This reflux will usually resolve following valveablation.[7] However, the high grades of reflux which our patient had tend to be associated with nonfunctioning, dysplastic kidneys. We did not obtain a pathologic diagnosis of renal dysplasia of the right kidney which had the high grade reflux. However, the radiologic studies are consistent with a dysplastic right kidney.

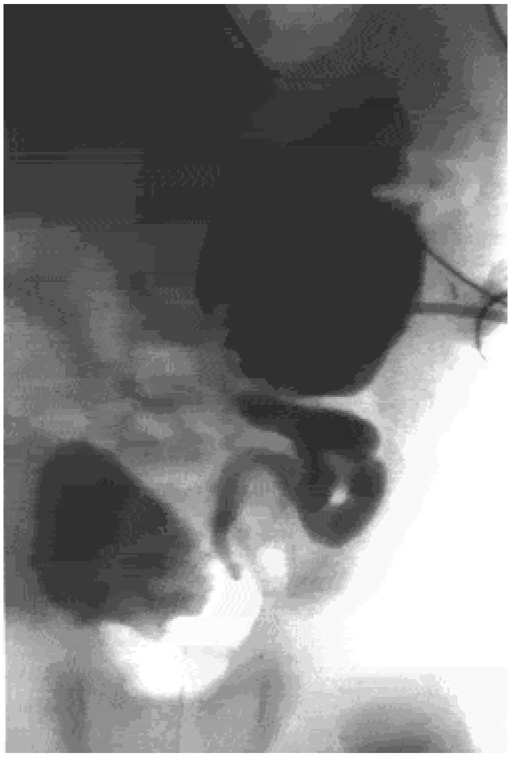

**FIGURE 4.** Left antegrade nephrostogram demonstrating non-obstructed flow into the bladder.

The exact cause of the VURD syndrome is unknown. However, Henneberry, Stephens, and Mackie theorized that it was not the reflux that caused the renal dysplasia but rather the abnormal position of the ureteric bud arising from the Wolffian duct.[5-7] The excessive lateralization of the ureteric bud from the mesonephric duct during embryological development leads to the formation of a peripheral portion of the metanephric blastema resulting in the development of a dysplastic renal unit.[4,5,7] The lower end of the ureter migrates into a more lateral position in the forming bladder and remains laterally ectopic with an inadequate submucosal tunnel that is too short to prevent reflux.[7]

Immediate treatment of the VURD syndrome consists of draining the urinary bladder through an indwelling urethral catheter (5 or 8 French feeding tube) and intravenous fluid therapy to correct electrolyte abnormalities, dehydration and metabolic acidosis. Serum creatinine should be closely observed. If the creatinine falls significantly below 1.0 mg/dl and there is only mild or moderate upper tract dilation in a relatively well baby, valve ablation should be considered.[5] Duckett found that patients with a nadir creatinine below 0.8 mg/ dl were unlikely to develop chronic renal failure and ESRD, end stage renal disease.[3] However, if the bladder is small and there is significant tortuosity of the ureters and hydronephrosis which was present in our patient, a simple valve resection might not be enough. Therefore, in such patients it is preferable to be assured that the bladder remains empty by employing a simple vesicostomy.[5]Worsening hydronephrosis and persistent elevation of serum creatinine in our patient prompted us to obtain a radionuclide renal scan to delineate the functional status of the kidneys. In such patients with worsening hydronephrosis and/or persistently elevated creatinine, temporary proximal diversion should be considered such as percutaneous nephrostomy tube, high ureterostomy, or cutaneous pyelostomy to assure adequate drainage from the renal unit.[3,5]

The decision to perform the upper tract intervention was based on the patient's elevated creatinine and worsening hydronephrosis in the left (functioning) renal unit. Some decline in the serum creatinine occurred after percutaneous nephrostomy whether the creatinine would have decreased further in this child is uncertain. However such prolonged intubated drainage carriers a real risk of infection and other complications, thus a non-intubated high urinary diversion was performed.

The other confounding consideration is the prematurity of the patient which can influence the outcome of transitional renal development. We felt that this patient had an excellent chance of obtaining normal renal function. However if the developing nephrons were subjected to obstruction as was evident with worsening hydronephrosis of the left (functioning) renal unit, renal injury as well as reduced glomerular filtration rate (GFR) would occur. After correction of this urinary obstruction, the premature infant's creatinine is expected to decrease as rapidly as a full term baby. [3]In our past experience in patients with similar nadir creatinines, we felt intervention, as described above, was most appropriate.

In conclusion our case demonstrated that in patients with VURD syndrome, worsening hydronephrosis and/or a persistently elevated creatinine may benefit from a high urinary diversion.
